# High-Resolution Microscopical Studies of Contact Killing Mechanisms on Copper-Based Surfaces

**DOI:** 10.1021/acsami.1c11236

**Published:** 2021-10-07

**Authors:** Tingru Chang, R. Prasath Babu, Weijie Zhao, C. Magnus Johnson, Peter Hedström, Inger Odnevall, Christofer Leygraf

**Affiliations:** † Department of Chemistry, Division of Surface and Corrosion Science, KTH Royal Institute of Technology, Drottning Kristinas väg 51, SE-100 44 Stockholm, Sweden; ‡ AIMES—Center for the Advancement of Integrated Medical and Engineering Sciences at Karolinska Institutet, KTH Royal Institute of Technology, SE-171 77 Stockholm, Sweden; § Department of Neuroscience, Karolinska Institutet, SE-171 77 Stockholm, Sweden; ∥ Department of Materials Science and Engineering, KTH Royal Institute of Technology, SE-100 44 Stockholm, Sweden

**Keywords:** contact killing, copper-based surfaces, Bacillus subtilis, bioinorganic interface, focused ion beam, transmission electron microscopy, nano-FTIR, intracellular particles

## Abstract

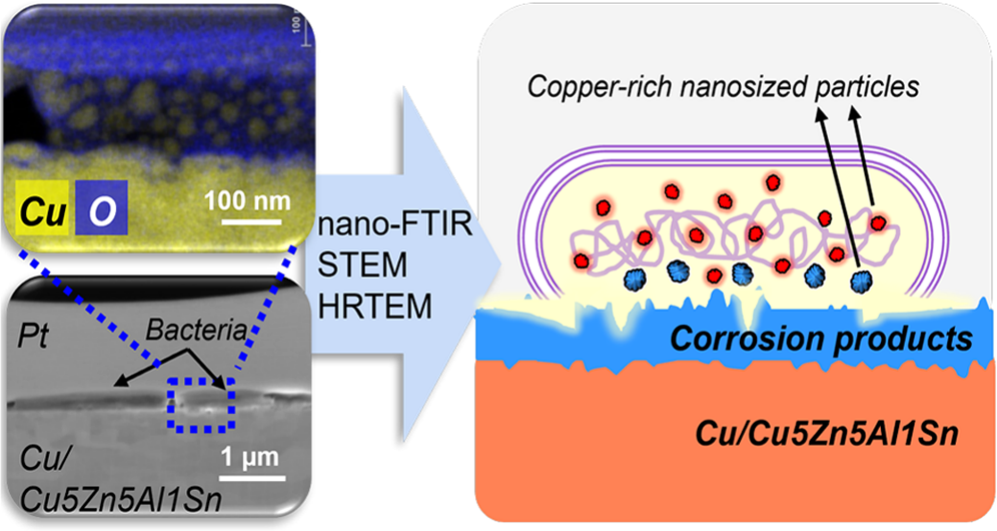

The mechanisms of bacterial contact killing induced by Cu surfaces were explored through high-resolution studies based on combinations of the focused ion beam (FIB), scanning transmission electron microscopy (STEM), high-resolution TEM, and nanoscale Fourier transform infrared spectroscopy (nano-FTIR) microscopy of individual bacterial cells of Gram-positive *Bacillus subtilis* in direct contact with Cu metal and Cu5Zn5Al1Sn surfaces after high-touch corrosion conditions. This approach permitted subcellular information to be extracted from the bioinorganic interface between a single bacterium and Cu/Cu5Zn5Al1Sn surfaces after complete contact killing. Early stages of interaction between individual bacteria and the metal/alloy surfaces include cell leakage of extracellular polymeric substances (EPSs) from the bacterium and changes in the metal/alloy surface composition upon adherence of bacteria. Three key observations responsible for Cu-induced contact killing include cell membrane damage, formation of nanosized copper-containing particles within the bacteria cell, and intracellular copper redox reactions. Direct evidence of cell membrane damage was observed upon contact with both Cu metal and Cu5Zn5Al1Sn surfaces. Cell membrane damage permits copper to enter into the cell interior through two possible routes, as small fragmentized Cu_2_O particles from the corrosion product layer and/or as released copper ions. This results in the presence of intracellular copper oxide nanoparticles inside the cell. The nanosized particles consist primarily of CuO with smaller amounts of Cu_2_O. The existence of two oxidation states of copper suggests that intracellular redox reactions play an important role. The nanoparticle formation can be regarded as a detoxification process of copper, which immobilizes copper ions via transformation processes within the bacteria into poorly soluble or even insoluble nanosized Cu structures. Similarly, the formation of primarily Cu(II) oxide nanoparticles could be a possible way for the bacteria to deactivate the toxic effects induced by copper ions via conversion of Cu(I) to Cu(II).

## 1 Introduction

The antimicrobial functionality of metals and alloys has gained increasing interest due to the emergence of antibiotic-resistant bacterial and virus strains that threaten vital health and result in economic challenges.^1−3^ Recent findings have highlighted the relatively rapid inactivation of SARS-CoV-2 on copper (Cu) and Cu-coated surfaces.^4−6^ Owing to the desirable antimicrobial efficiency, Cu metal and Cu-based alloys surfaces are increasingly used as promising high-touch surfaces for hygienic applications to reduce the occurrence of healthcare-associated infections.^7−10^ The ability of Cu metal and Cu-based alloys to inactivate or kill 99.9% pathogenic bacteria within 2 h has been certified by the U.S. Environmental Protection Agency (EPA).^11^ As a result of the considerable reduction in the number of bacteria, the concept of “contact killing” induced by Cu and Cu-based alloys has been introduced. It refers to lethal bacterial damage in contact with the metallic surface.^12,13^ Even though the underlying main mechanism is not yet established, contact killing has been largely related to the release of copper ions from Cu-based surfaces.^14−17^ Reported mechanisms for the antimicrobial efficiency of Cu involve cell membrane damage, protein inactivation, decay of DNA function, and suppression of respiration.^7,18^

During use as high-touch surfaces at indoor atmospheric conditions, copper(I) oxide (cuprite, Cu_2_O) is inevitably formed on any Cu or Cu-alloy surface,^19^ sometimes mixed with copper hydroxychloride (Cu_2_(OH)_3_Cl) caused by finger imprint.^20^ More complex corrosion products may also form depending on the prevailing environmental conditions, e.g., during exposure to human palm sweat.^21,22^ A recent study showed that the influence of the formed corrosion products on the antimicrobial property (against *Bacillus subtilis*, Gram-positive) is different for Cu and Cu5Zn5Al1Sn (the Golden Alloy, containing approximately 5 wt % Zn, 5 wt % Al, and 1 wt % Sn).^23^ No significant change was observed for the antimicrobial effects of Cu metal surfaces before and after a short-term indoor atmospheric exposure (i.e., both surfaces exhibited rapid contact killing within minutes), whereas an enhanced antimicrobial efficiency was observed on the more corroded Cu5Zn5Al1Sn surface during the initial contact due to higher concentrations of released copper and zinc ions.^23^ These differences in antimicrobial efficiency and copper-ion release between Cu and Cu5Zn5Al1Sn were mainly attributed to different surface compositions and physicochemical properties of the corrosion products formed. A considerably thinner and more compact corrosion product layer with enhanced barrier properties was observed on Cu5Zn5Al1Sn than on Cu,^24,25^ resulting in lower copper-ion release rates and an overall initially slightly slower contact killing rate than observed for Cu metal.^23^ The same study further showed that the reduced antimicrobial property of Cu5Zn5Al1Sn, i.e., a slightly higher initial viability of bacteria (*B. subtilis*) on the Cu5Zn5Al1Sn surface during the initial contact of approximately 6 min, leads to an enhanced formation of bacteria-produced extracellular polymeric substances (EPSs) on the surface. With increased time, the EPSs develop further into a biofilm, which alters both the surface chemistry and the corrosion resistance.^23^ However, the influence of corrosion products formed on Cu metal and Cu5Zn5Al1Sn on surface-adherent bacteria is unknown and has never been investigated with respect to rapid contact killing. A more detailed state-of-the-art characterization of the bioinorganic interface between bacteria and Cu/Cu5Zn5Al1Sn surfaces is still lacking.

The aim of the current study is to provide an insight into the underlying mechanisms of copper-induced contact killing through high-resolution surface and interface investigations by elucidating the interaction between bacteria and corrosion products formed under simulated indoor high-touch conditions of Cu metal and Cu5Zn5Al1Sn surfaces. The laboratory simulation of indoor high-touch conditions and the resulting antimicrobial properties of the Cu/Cu5Zn5Al1Sn surfaces investigated are given in our preceding publications.^20,23^ The laboratory simulation is based on preoxidized and corrosion of high-touch surfaces through artificial sweat deposition and subsequent dry/wet cycling, with further details given in the Experimental Section. The strain, *B. subtilis* (Gram-positive), used in the current study and a previous study,^23^ is capable of forming complex bacteria communities in early and later stages of a biofilm,^26^ which is mainly composed of EPSs.^27^ This facilitates the study of the possibly intertwined effect of corrosion products and organic residues produced by the bacteria. It should be emphasized that the mechanistic insight gained from the present study, with a Gram-positive bacterium (*B. subtilis*) in focus, may not be representative for other types of bacteria because of the specificity of interfacial mechanistic processes involved. Nevertheless, we believe that the present high-resolution microscopy approach may be appropriate for mechanistic studies of contact killing of other bacteria as well.

In the current work, the topography of adherent bacteria on Cu/Cu5Zn5Al1Sn surfaces was characterized by focused ion beam-scanning electron microscopy (FIB-SEM), followed by an analysis of a selection of individual bacteria and the adjacent Cu/Cu5Zn5Al1Sn surfaces through FIB milling. Each FIB lamella was lifted out and transferred to a TEM grid for ultrastructural cell imaging with scanning transmission electron microscope (STEM) analysis, aiming at detailed studies of the interaction between individual bacteria and the metallic surface constituting a bioinorganic interface. In addition, topography changes of individual bacteria in contact with the Cu/Cu5Zn5Al1Sn surfaces and their concomitant surface chemistry changes induced by contact with individual bacteria were analyzed by means of nanoscale Fourier transform infrared spectroscopy (nano-FTIR). This combination of advanced analytical tools permits new aspects to be revealed of the complex interplay between bacteria and the adjacent surfaces of metals/alloys.

## 2 Experimental Section

### 2.1 Materials and Surfaces

In-depth studies were performed on coupons of bare Cu metal (DHP-Cu,^28^ purity 99.98%) and a commercially available copper alloy (Cu5Zn5Al1Sn: 89 wt % Cu, 5 wt % Zn, 5 wt % Al, ∼1 wt % Sn), which were kindly provided via the international copper industry. As-received Cu and Cu5ZnAl1Sn surfaces were consecutively ground to #4000 grit using silicon carbide paper followed by ultrasonic cleaning in analytical-grade ethanol for 5 min. The surfaces were subsequently dried by cold nitrogen gas followed by the uniform deposition of artificial sweat (ASW, mean deposited mass of 6.75 ± 0.77 mg/cm^2^) using an airbrush. More detailed information is given in ref (20). The ASW used in this study was prepared according to the EN 1811 standard^29^ and was always freshly used within 1 day of preparation. ASW is composed of 5.0 g/L sodium chloride (NaCl), 1.0 g/L urea (CH_4_N_2_O), and 1.0 g/L lactic acid (C_3_H_6_O_3_) dissolved in ultrapure water (Milli-Q, 18.2 MΩ·cm). The pH of ASW was adjusted to 6.5 ± 0.05 by adding NaOH. All of the chemicals used here were purchased from VWR chemicals and Sigma-Aldrich (Sweden).

Cu and Cu5Zn5Al1Sn coupons with and without predeposited ASW were immediately transferred to a climatic chamber followed by wet/dry cyclic exposures at 25 °C to mimic the skin contact situation under high-touch-induced atmospheric corrosion conditions.^20,30^ It is to be noted that 1 day of wet/dry exposures includes 4 h of wet (RH 90%) and 2 h of dry (RH 0%) conditions followed by another 16 h at wet (RH 90%) and 2 h of dry (RH 0%) conditions. Three coupons with different extents of corrosion were selected for further investigations, i.e., Cu metal with and without predeposited ASW, and Cu5Zn5Al1Sn with predeposited ASW, each followed by 1 day of cyclic wet/dry exposures. From now on, these samples are denoted Cu, Cu_ASW_, and Cu5Zn5Al1Sn_ASW,_ respectively.

### 2.2 Bacteria Preparation and Viability Analysis

A *B. subtilis* (ATCC 23857) culture was grown overnight in nutrient broth no. 4 (Sigma-Aldrich, Sweden) at 30 °C. The overnight culture was washed two times and resuspended in an ASW solution followed by dilution to a bacterial concentration of OD_600_ = 0.05. OD_600_ is the spectrophotometrically determined optical density at a wavelength of 600 nm.

**Figure 1:**
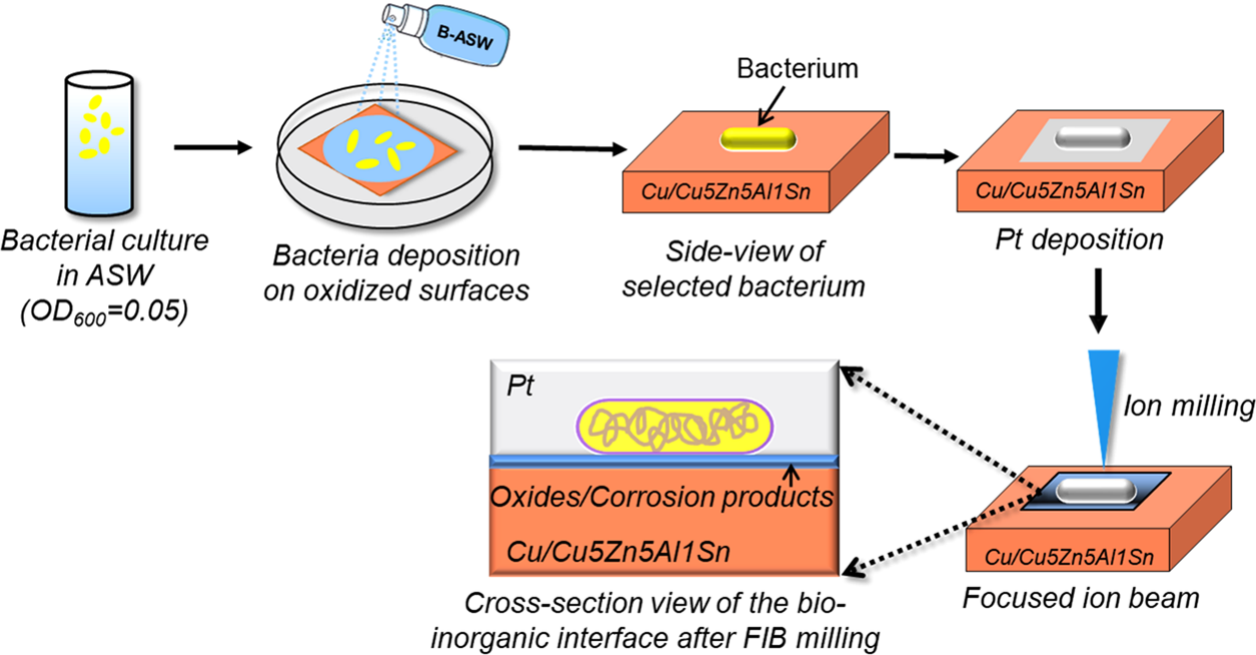
Schematic illustration of the bacteria culture, surface preparation, and the approach for the Cu/Cu5Zn5Al1Sn–bacterium interfacial analysis.

The bacterial culture (OD_600_ = 0.05) were deposited onto the Cu, Cu_ASW,_ and Cu5Zn5Al1Sn_ASW_ surfaces, respectively, using the same method as in a previous study to simulate high-touch conditions. This procedure was shown to realistically mimic high-touch conditions.^20,23^ The preparation of ASW containing bacteria, the bacteria deposition, and the following interface preparation are schematically illustrated in Figure 1. Bacteria-containing ASW was deposited six times onto the Cu and Cu5Zn5Al1Sn surfaces with 10 min interval times between each deposition to ensure that each deposition was made on a relatively dry surface. See ref (23) for details of the viability tests.

### 2.3 Surface and Interface Analysis

#### 2.3.1 Surface Composition

Nano-FTIR microscopy [scattering-type scanning near-field optical microscope (s-SNOM), Neaspec GmbH, Germany] equipped with atomic force microscopy (AFM) for topography imaging and a broad-band laser for nanoscale IR spectroscopy studies was used to identify the functional groups of a single bacterium interacting with a Cu5Zn5Al1Sn surface.^31^ The probing depth under current conditions is in the order of tens of nm.^31^ The nano-FTIR AFM tips from Neaspec had a diameter of about 50 nm. The back-scattered light was collected using a mercury cadmium telluride (MCT) detector and analyzed using an asymmetric Michelson interferometer with a spectral resolution of 30 cm^–1^ and a recording time of 200 s for each spectrum. The spectra acquired from the bacterium area were normalized to the spectra from the same surface without any adherent bacteria. The AFM images were processed using software Gwyddion.^32^ Further details of nano-FTIR microscopy are given in ref (31).

#### 2.3.2 Surface Morphology and Interface Preparation

The surface topography of the precorroded Cu coupons after bacteria deposition was analyzed by means of an FEI (ThermoFisher) Nova600 FIB-SEM at an accelerating voltage of 2 kV. One or two bacteria were selected from each coupon for the investigation of Cu/Cu5Zn5Al1Sn–bacterium interfaces. The surfaces of the selected areas of interest were protected by platinum deposition first in electron beam and then an ion beam to a final thickness of about 2 μm. Cross sections were made in the longitudinal direction of the selected bacteria to ensure the availability of larger interface areas for further analyses. This process is also schematically illustrated in Figure 1. Thin lamellae were produced by the Ga-ion beam milling and lifted out in situ by an Omniprobe micromanipulator in the FIB-SEM. The lifted out lamellae were mounted on a molybdenum grid with Pt welding and further thinned down to a ca. 150 nm thickness. This mounted lamella was used for transmission Kikuchi diffraction (TKD) and was further thinned down to ca. 50 nm for TEM analysis. The final stage of ion milling was carried out at a voltage of 30 kV and a current of 30 pA followed by a cleaning step at 5 kV.

#### 2.3.3 Interface Characterization

The subcellular information of the selected Cu/Cu5Zn5Al1Sn–bacteria interfaces in terms of morphology, structure, and chemical distribution were investigated with a probe Cs-corrected scanning transmission electron microscope (STEM) Titan Themis (ThermoFisher) at 200 kV equipped with a super-X energy-dispersive X-ray spectrometer (EDXS). The crystalline lattice structure of the Cu-containing features within the bacteria was characterized by means of high-resolution TEM (HRTEM). TEM bright-field, STEM bright-field, and high-angle annular dark-field (HAADF) imaging were used to characterize the morphology and the structure of the metal–bacteria interfaces. Chemical information was obtained as spectral maps through EDXS measurement with a Bruker Esprit 1.9 interface (Germany).

The microstructure and crystallographic information of the interfacial region between the Cu/Cu5Zn5Al1Sn surfaces and bacteria were investigated by means of TKD in an FIB-SEM Nova600 with an acceleration voltage of 20 kV and a beam current of 2.4 nA. An Oxford Instrument’s Symmetry CMOS detector was utilized for this data acquisition through the AZTEC 4.3 user interface. Data analysis was performed with AZTEC Crystal software.

## 3 Results and Discussion

To gain new mechanistic insight into contact killing, the bioinorganic interface between a single bacterium and differently oxidized/corroded Cu or Cu5Zn5Al1Sn surfaces used for high-touch surface conditions was investigated using a multianalytical approach.

### 3.1 FIB-SEM Topography and Nano-FTIR Composition Analyses

**Figure 2:**
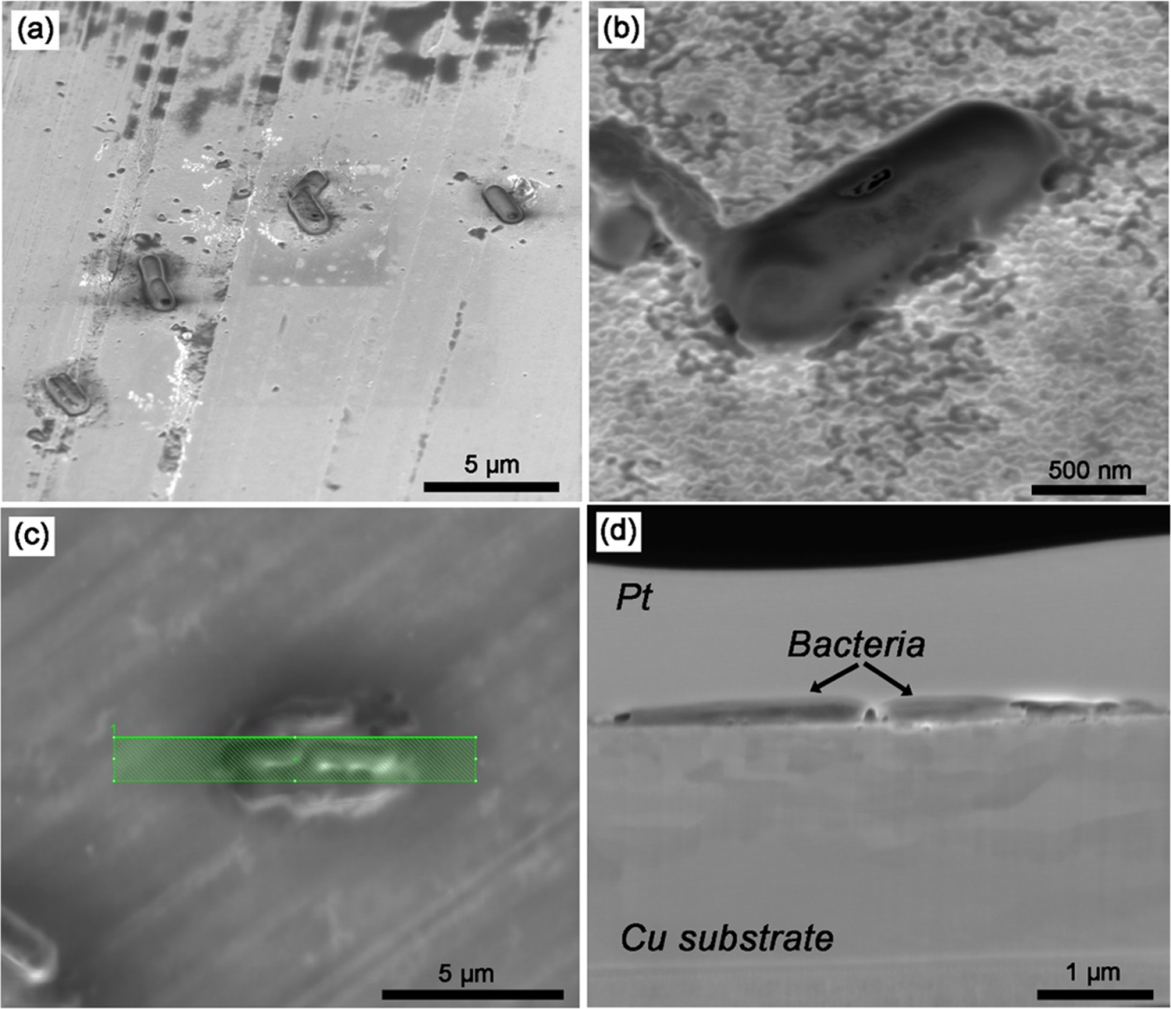
FIB-SEM morphologies of bacteria on a preoxidized Cu surface without ASW: oblique view (a, b), vertical view of the selected bacterial cells for ion milling (c), and cross-sectional view of the bacteria–Cu interface after Pt deposition and ion milling (d).

The surface morphology of the preoxidized Cu coupon without predeposited ASW was first investigated with FIB-SEM, and is displayed in the oblique view in Figure 2a,b. The images show adherent bacteria on the Cu surface. Some small pits and dark gray spots were observed adjacent to the bacteria, indicating that local areas of the Cu surface surrounding the bacterium were altered. A closer observation of one of the bacteria, Figure 2b, shows dark gray matter emanated from the bacterium that was diffusing outward along the surface oxide of the Cu surface. These features suggest interactions between the bacteria and the surface oxides. A cross section of the bacteria–Cu interface was prepared next by means of FIB for in-depth analysis, and two bacteria in the marked zone shown in Figure 2c were selected for the FIB preparation. The cross-sectional view of the bacteria–Cu interface after FIB milling is displayed in Figure 2d. Due to the limited spatial resolution of FIB-SEM, the explicit characteristics of this interface, including the subcellular structure and the corresponding chemical information, were studied in more detail by TEM, as described in Section 3.2.

**Figure 3:**
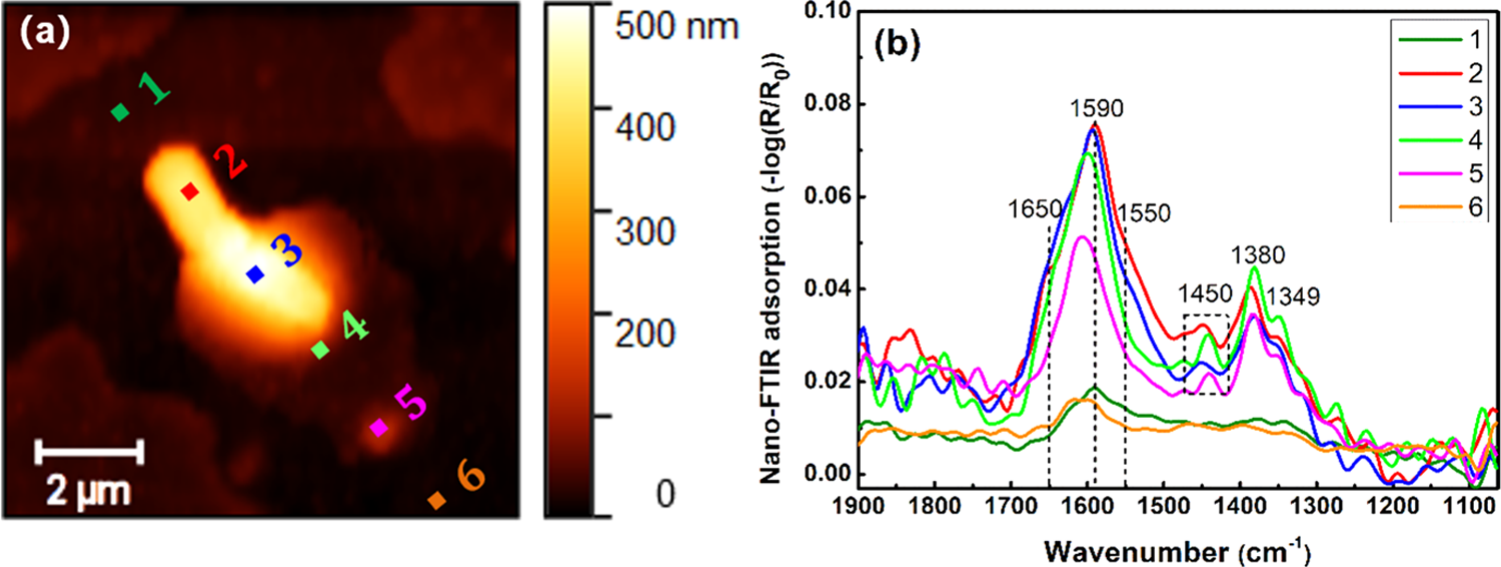
AFM topography image (a) and corresponding nano-FTIR spectra (b) obtained from a selected area within and adjacent to an individual bacterium in contact with a polished Cu5Zn5Al1Sn surface.

Prior to the more detailed interface characterization by TEM, a bacterium adhering to a Cu5Zn5Al1Sn surface was investigated by AFM-based nano-FTIR microscopy with respect to surface topography and chemical composition. Nano-FTIR was conducted on polished Cu5Zn5Al1Sn due to instrumental requirements of low surface roughness to follow surface compositional changes along a single bacterium with a spatial resolution of about 50 nm. The AFM image of the individual bacterium and IR spectra collected from the corresponding zones in the AFM image are presented in Figure 3a,b, respectively. The size of the investigated bacterium is approximately 4–5 μm in length, 1 μm in width, and 0.5 μm in height, as shown in Figure 3a. The topography suggests an impaired bacterial cell membrane in contact with the polished Cu5Zn5Al1Sn surface, implying bacterial death under these conditions. IR spectra 2 and 3 were acquired from the areas within the cell membrane (including outer membrane and cytoplasmic membrane in this study), spectra 4 and 5 from the periphery area containing released substances from the bacterium, and spectra 1 and 6 from areas outside the bacterium. Strong and broad bands between 1700 and 1500 cm^–1^ observed in spectra 2–4, of lower intensity in spectrum 5 and substantially lower intensity (almost absent) in spectra 1 and 6 suggest the presence of amides (shoulders at 1650 cm^–1^ for amide I and at 1550 cm^–1^ for amide II).^33,34^ The most intense band at approximately 1600 cm^–1^ can be assigned to the antisymmetric stretch of COO^–^,^35^^35^ overlapping with the amide bands.^36^ These bands originate most likely from peptidoglycan (saccharides and amino acids) and proteins within the cell envelope, i.e., either from the cytoplasmic membrane or from other components in the cell.^37^ Bands within the region between 1470 and 1300 cm^–1^ are primarily due to bending vibrations of −CH_3_, −CH_2_, and symmetric stretching of COO^–^ groups from proteins and polysaccharides,^34,38^ indicating the leakage of cellular substances and/or the generation of EPSs on and around the bacterium. Spectra 4 and 5 show higher intensities of the band between 1460 and 1420 cm^–1^ than spectra 2 and 3, where the band close to 1450 cm^–1^ is due to the symmetrical deformation vibrations of C–OH and CH_2_.^36,39^^36,39^ Observed shifts of the band at 1450 cm^–1^ in spectra 2–5 are probably caused by the interaction of organic species with the Cu5Zn5Al1Sn surface.^39,40^

To conclude, the AFM image provides evidence of cell membrane damage upon contact with the Cu5Zn5Al1Sn surface. Associated nano-FTIR spectra reveal changes in surface chemistry upon the interaction between the bacterium and the alloy substrate and evidence of cell leakage from the bacterium, which suggest changes in the surface composition induced by the cell leakage. Cell ruptures most likely also take place on a Cu surface, for which slightly higher instantaneous killing rates of *B. subtilis* were observed (though not statistically proven) compared with Cu5Zn5Al1Sn.^23^ In all, adhered bacteria change the surface composition at the Cu/Cu5Zn5Al1Sn–bacterium interface.

### 3.2 TEM Analyses of Bioinorganic Interfaces

**Figure 4:**
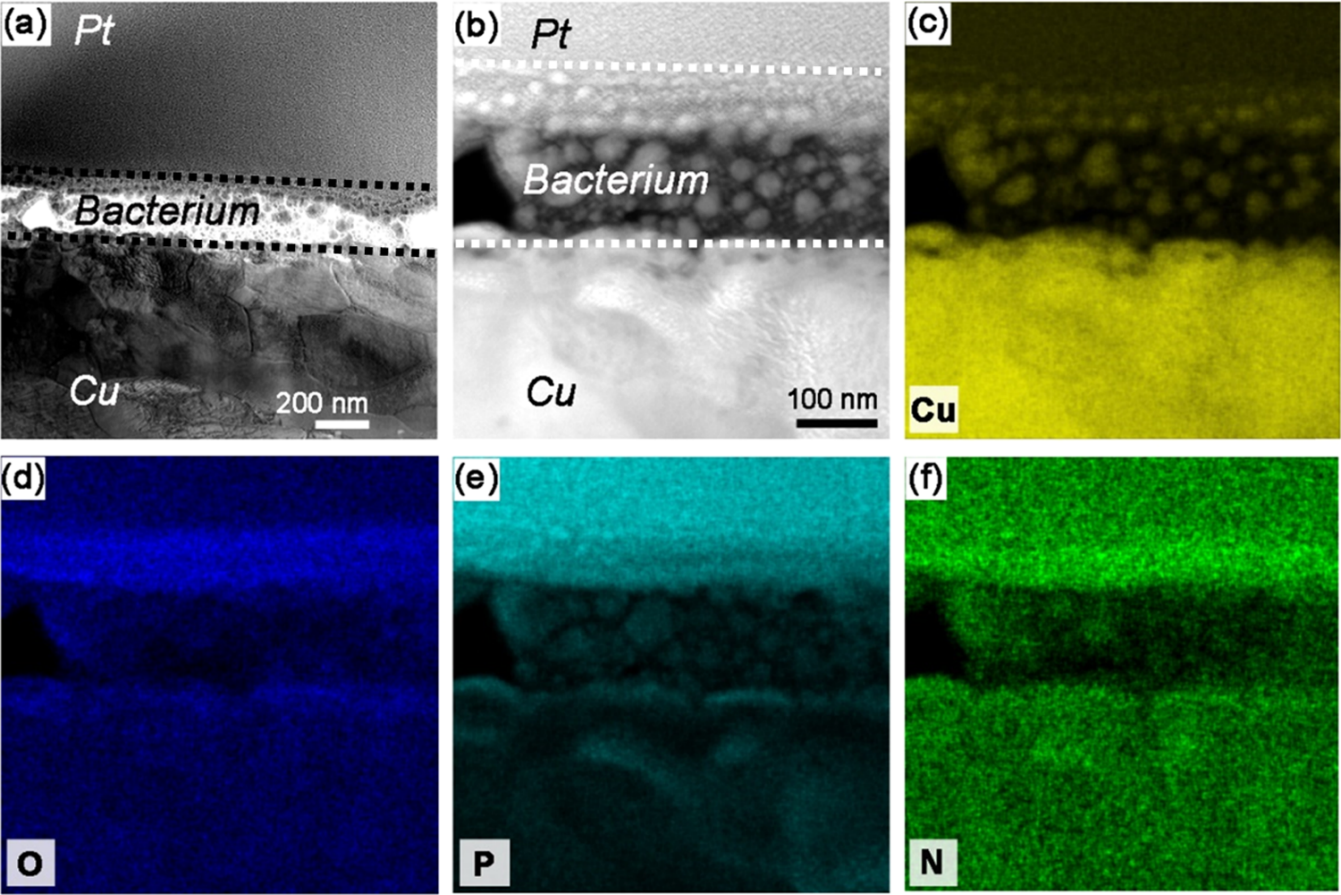
STEM bright-field (a) and enlarged HAADF (b) images of the interfaces between the Pt protection coating, bacterium, and Cu (from up to down) and the corresponding STEM–EDS maps of Cu (c), O (d), P (e), and N (f). The dashed lines in (a) and (b) just roughly indicate the location of the bacterium as the interface between bacterium and Cu cannot be unambiguously distinguished.

**Figure 5:**
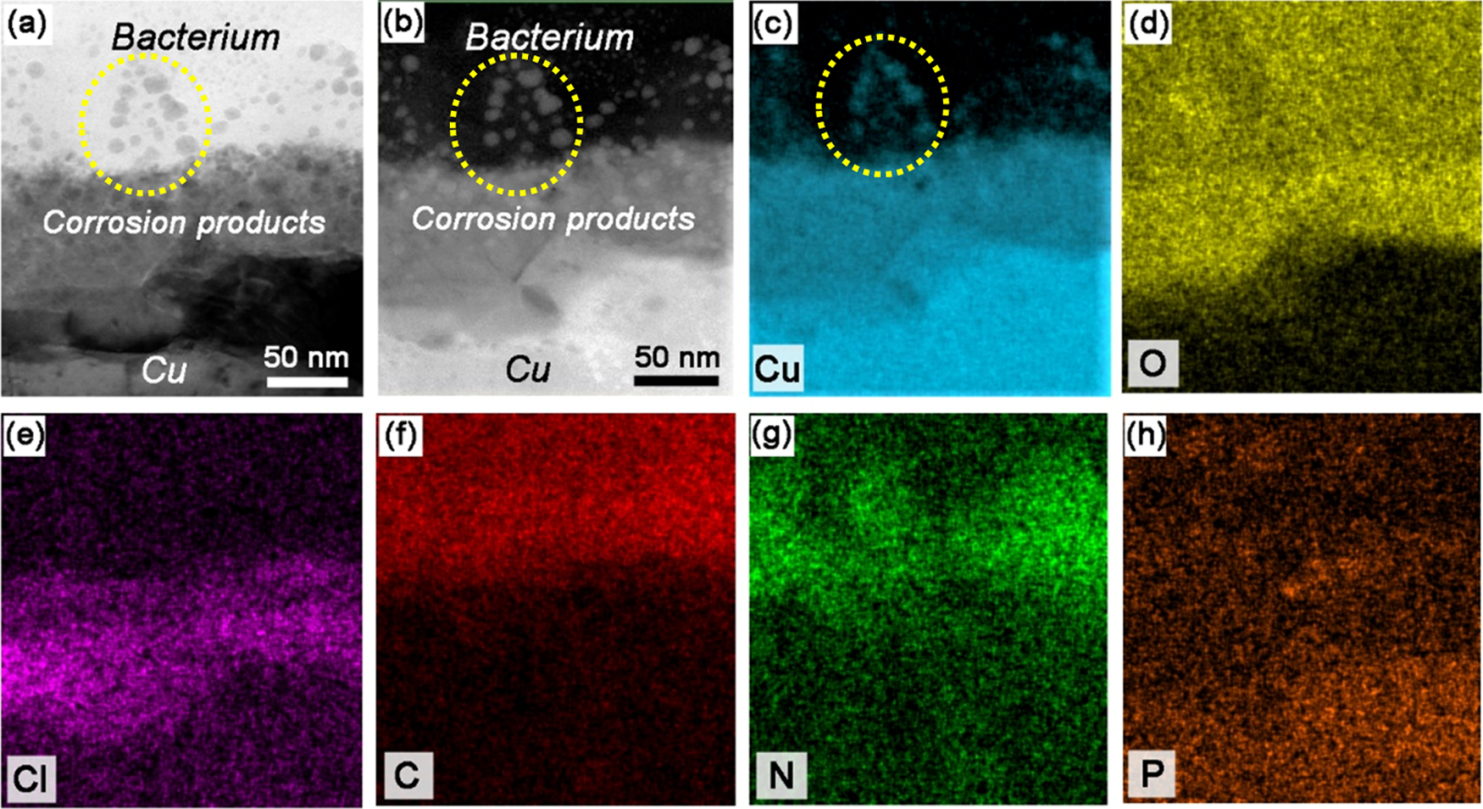
STEM bright-field (a) and enlarged HAADF (b) images of interface morphology between a bacterium and a Cu_ASW_ surface (left, middle on the top) and the corresponding STEM–EDS maps of Cu (c), O (d), Cl (e), C (f), N (g), and P (h).

The bioinorganic interface between the Cu surface and the adhered bacterium shown in Figure 2d was analyzed in more detail by STEM. Figure 4a,b shows the bright-field and HAADF images of selected areas, while the corresponding elemental distributions of Cu, O, P, and N are displayed in Figure 4c–f. The corresponding results from the bioinorganic interface for the Cu_ASW_ (i.e., with predeposited ASW) surface characterized by STEM is shown in Figure 5 for comparison, with the bright-field and HADDF images in Figure 5a,b, respectively, and the corresponding elemental distributions of Cu, O, Cl, N, and P in Figure 5c–h.

Individual grains in the Cu substrate can be observed in Figure 4a. The bacterial cell exhibits an approximate total thickness (diameter) of 200 nm, which is thinner than *B. subtilis* in its stationary phases (0.25–1 μm).^41,42^ The thinner bacterial cell, in this case, is probably due to cell dehydration once the bacterium was in contact with the Cu surface, exposed to the high vacuum conditions of TEM/STEM characterization, and during sample preparation in FIB-SEM. It is also seen in Figure 4b that the cell membrane structure at the lower side in contact with the Cu surface is absent, while the upper side in contact with the Pt coating is relatively intact. This implies that the cell structure was damaged once in contact with the Cu surface, which is probably one reason for contact killing of copper.^7^

An important observation in Figure 4b is the evidence of nanosized particles within the cell and the cell membrane. Their corresponding elemental distributions in Figure 4c–f illustrate that copper is enriched within these particles as well as within the cell membrane, indicating the admittance of copper into the interior of the cell and the cell membrane. This may be a consequence of rupture of the cell membrane. An enrichment of O, P, and N is also observed in the intact cell membrane, as shown in Figure 4d–f. The enrichment of O at the interface between the bacterium and the Cu surface is evidence of surface oxide and/or a layer of cell-released substances (e.g., EPSs, biosurfactants). When comparing the distribution of O with the distributions of P and N, it is seen that the enrichment of O occurs both within a thin layer of cell-related matter, enriched in N and P, and the thin layer of surface oxide next to the Cu surface. The presence of P and N can also be seen beneath the copper oxide layer further into the Cu substrate, which probably suggests the penetration of bacteria residues into the Cu substrate, although the artifacts due to FIB milling cannot be excluded. This is evidence of the complex interplay between the bacterium and the copper surface, as also seen from different perspectives with SEM (Figure 2a,b, cross-sectional view) and nano-FTIR (Figure 3, top view). A possible reason may be the leakage of reducing saccharides and amino acids from the disrupted bacterial cell membrane^17^ and of peptidoglycans into the Cu oxide and the adjacent Cu substrate. Peptidoglycans consist of a polymeric substance of sugars and amino acids and constitute a major part of the cell membrane in Gram-positive bacteria.^43^

Figure 5a,b displays three distinguishable layers at the bacterium–Cu_ASW_ interface. From top to bottom they correspond to the bacterium, corrosion products, and the Cu substrate, with the corresponding elemental distributions seen in Figure 5c–h. The cross-sectional morphology of Cu_ASW_ without a bacterium is displayed in Figure S1 to demonstrate the morphological difference of the corrosion products layer with and without the bacterium. The absence of a clear cell membrane structure in Figure 5a,b between the bacterium and the Cu substrate suggests that the cell membrane, in this case, was also damaged in contact with the more corroded Cu surface. A thicker layer of corrosion products can be seen after predeposition of ASW (Figure 5) than without (Figure 4). This layer is enriched with Cu, O, and Cl and consists mainly of Cu_2_O, Cu_2_(OH)_3_Cl, and/or CuCl, according to our previous studies.^20,23^ Similar to the bacterium in contact with the less corroded Cu, Figure 4, nanosized copper-rich particles can also be clearly observed within the cell in contact with this more corroded Cu surface (Cu_ASW_). The particles are distributed in the volume closer to the corrosion product layer, as indicated by dotted circles in Figure 5a–c. The enrichment of N within the area containing these particles, Figure 5g, suggests the binding of N-containing biomolecules (e.g., amino acids) with copper ions. Similar enrichments of N can be also found at the interfaces between the cell and the Cu/Cu5Zn5Al1Sn_ASW_ surface (see Figures S2 and S3). This overlap between N and Cu within the cell may possibly be caused by copper displacement of iron from iron–sulfur clusters of dehydratases^44^ and by copper ions, which compete with other metal ions for crucial binding sites on amino acids/proteins.^7^

To conclude, the STEM–EDS results suggest that the rupture of the cell membrane in contact with the Cu surface and the formation of copper-rich nanosized particles inside the cell and in the cell membrane are pronounced features of contact killing of Cu. These features are seen on both Cu metal and, to a lesser extent, on Cu5Zn5Al1Sn_ASW_, and are most probably related to a thinner layer of corrosion products on the latter surface. The copper-rich particles are associated with the enrichment of N, which possibly is a consequence of cell damage and the concomitant complexation between copper ions and biomolecules.

**Figure 6:**
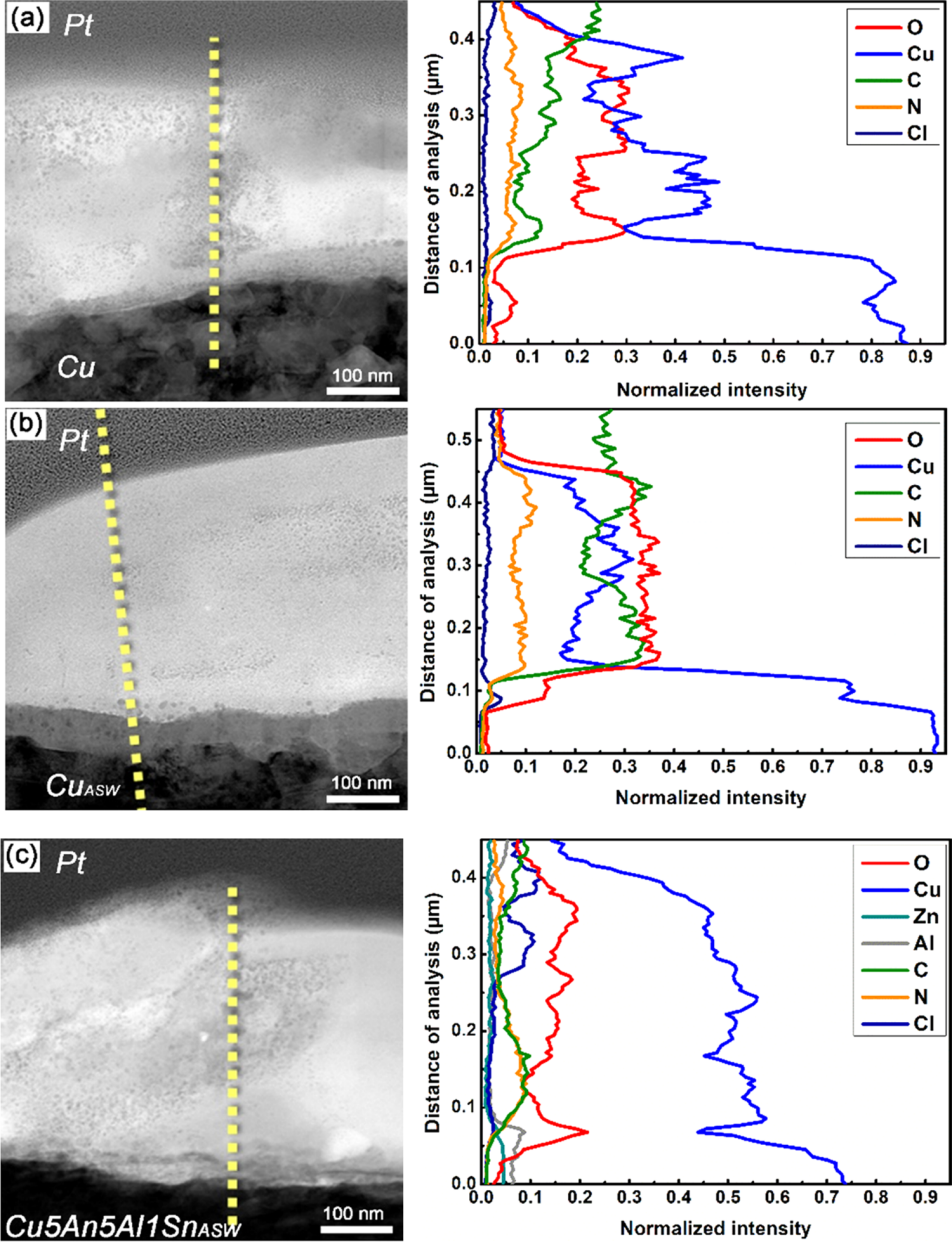
Bright-field STEM images (left) of Pt coating-bacterium–Cu surface (a), −Cu_ASW_ (b), and −Cu5Zn5Al1Sn_ASW_ (c) interfaces (from top to bottom) and the corresponding elemental distributions obtained by STEM–EDS line analysis (right).

Line analyses by means of STEM–EDS were conducted on all three bioinorganic interfaces, i.e., bacteria in contact with Cu, Cu_ASW_, and Cu5Zn5Al1Sn_ASW_ surfaces, respectively, to obtain more detailed elemental distributions, as shown in Figure 6a–c. Nanosized particles were observed in greater abundance within the bacterium in contact with Cu than in contact with Cu_ASW_ and Cu5Zn5Al1Sn_ASW_. This may be related to the higher extent of copper-ion release from Cu than from the other two surfaces.^20,23^ The EDS results also show that copper was distributed within the bacterial cell and present in all three interfacial regions. The same observation is made for O, C, and N, although their enrichment distributions are different with a higher enrichment within the cell in contact with the surfaces of Cu and Cu_ASW_ than with Cu5Zn5Al1Sn_ASW_. This implies that the extent of intrusive copper in contact with Cu5Zn5Al1Sn interacting with various cell constituents (e.g., proteins, lipids) within the bacterium was less compared with corresponding observations made on Cu surfaces because of less corrosion and lower concentrations of dissolved copper. It should be emphasized that it is not possible to unambiguously identify the nature of the biomolecule–copper interaction. Nevertheless, the possible underlying mechanism will be further discussed, based on available literature information.

**Figure 7:**
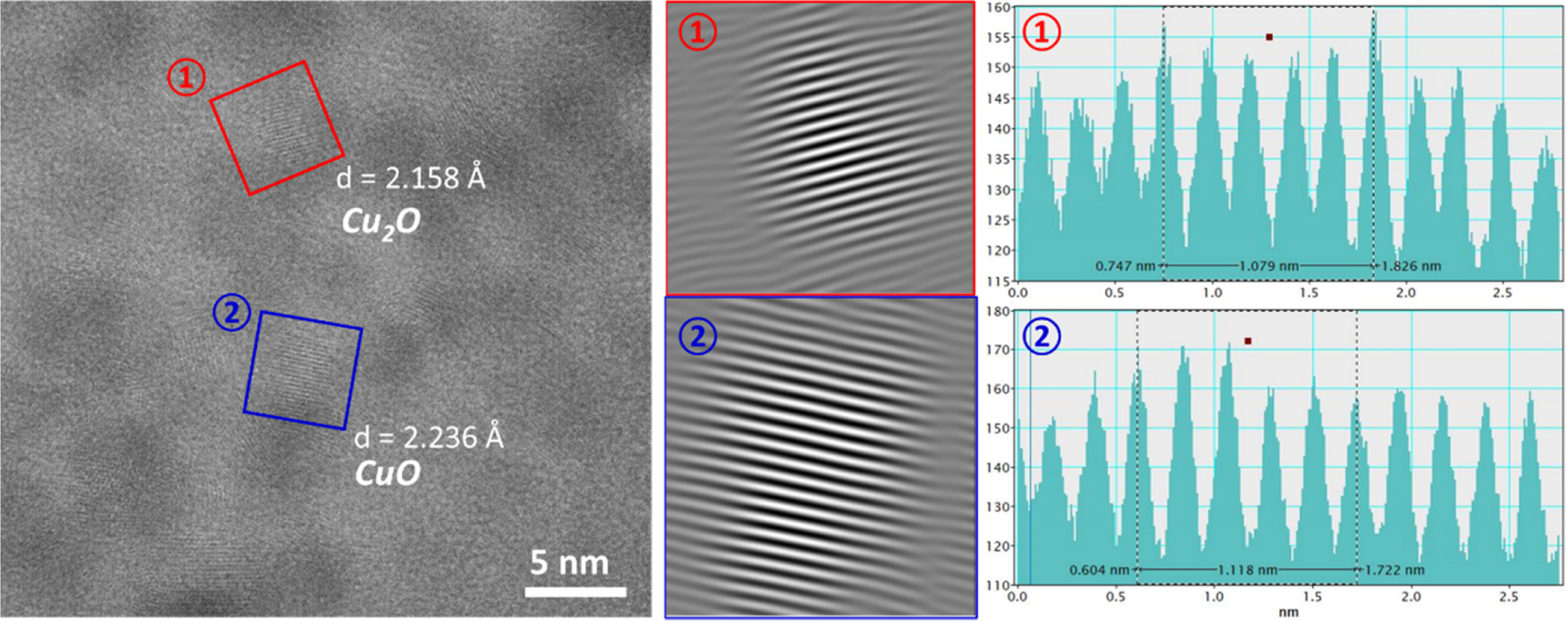
HRTEM image (left) of the copper-enriched particles found within the bacterium in contact with the Cu surface, the corresponding inverse fast Fourier transform (IFFT) image (middle), and the line profiles (right) generated from indicated areas of the HRTEM image.

The intracellular copper-containing nanosized particles were further identified by means of HRTEM. One selected area of analysis within the cell in contact with the Cu surface is displayed in Figure 7. The results of HRTEM demonstrate the crystalline structure of these particles. The values of the lattice spacings of the particles in the cell obtained through the line profile analysis suggest that the copper-rich particles consist of Cu_2_O (2.135 Å, (002)) and CuO (2.246 Å, (201̅)), rather than metallic Cu. The presence of crystalline copper oxides is also seen in particles inside the cells in contact with Cu_ASW_ and Cu5Zn5Al1Sn_ASW_, as shown in Figure S4. Thus, the HRTEM results suggest that the formation of these copper-rich nanosized particles within the cells are either due to the ingress of copper oxides formed at the Cu surface (mainly Cu_2_O rather than CuO) into the cells or the oxidation of copper ions dissolved from the surface/surface oxides into the cells. The different possibilities of the origin of these copper oxide nanoparticles in the cell reflect, as will be discussed later, different mechanisms of Cu killing.

### 3.3 TKD Characterization of Bioinorganic Interfaces

**Figure 8:**
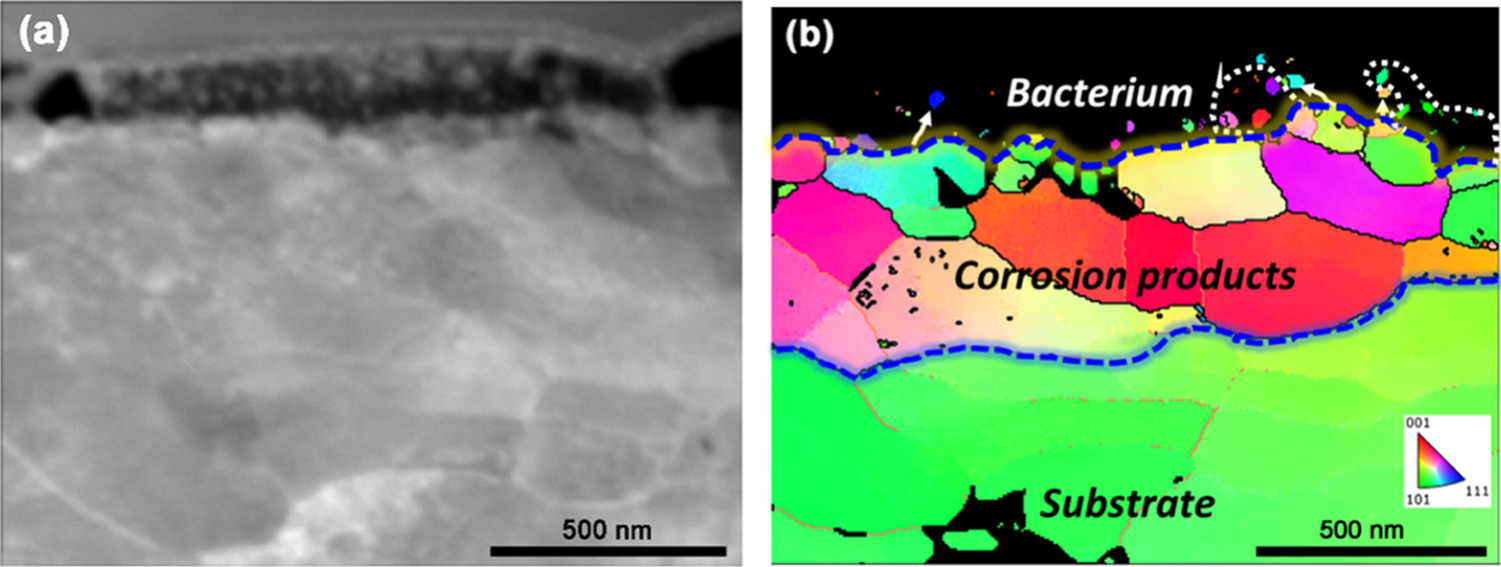
SEM morphology of the interface between a bacterium and the Cu surface (up to down) (a), TKD inverse pole figure (IPF) map (b) with indications of high- (misorientation > 15°) and low- (5° < misorientation < 15°) angle boundaries in black and orange lines.

Since the copper-containing nanoparticles exhibit a crystalline structure, the FIB-prepared bioinorganic interfaces were also analyzed by TKD to obtain microstructural information of the Cu/Cu5Zn5Al1Sn surface with corrosion products that could possibly provide additional information of the bacterium–Cu surface interface. The overview morphology and TKD maps of the bacterial cell–Cu interface are shown in Figure 8. The boundary between the Cu substrate and the corrosion product layer can be distinguished by the high-angle boundaries (misorientation > 15°) and significantly smaller grain size of the corrosion product layer than the Cu matrix of an average value of 10.4 μm,^24^ which is indicated by the blue dashed line in the inverse pole figure (IPF) map. The boundary between the corrosion product layer and the bacterium is also indicated in Figure 8b to roughly distinguish the small grains within the bacterium. It depicts that some of the larger particles can be indexed by TKD, revealing similar grain orientations as the adjacent grains of the oxide layer, see white indication circles and arrows in Figure 8b. This suggests that the larger particles close to the oxide layer are fragmentized from the oxide formed on the surface. Similar release of Cu oxide particles is also evident at the bacterium–Cu_ASW_ interface, as shown in Figures 5 and 6b. The ingress of these relatively larger particles into the bacterium may be a consequence of cell membrane damage. These results further verify the interplay between the bacterium and the corrosion products formed under simulated high-touch conditions. On the other hand, no particles could be indexed by TKD within the bacterium in contact with Cu5Zn5Al1Sn_ASW_, as shown in Figure S5. This is probably a consequence of thinner and more compact oxides/corrosion products present on the Cu5Zn5Al1Sn_ASW_ surface.^24,25^

### 3.4 Discussion on Mechanisms of Cu Contact Killing

The release/dissolution of copper ions from the three surfaces investigated herein, Cu, Cu_ASW_, and Cu5Zn5Al1Sn_ASW_, was triggered by depositing 3 μL of ASW-containing drops during 10 min contact exposure of each surface after 1 day of wet/dry aging. The measured quantities of released copper ions under these conditions were 0.070, 0.050, and 0.042 μg/cm^2^ from the Cu, Cu_ASW_, and Cu5Zn5Al1Sn_ASW_ surfaces, respectively; see refs^20, 23^ for further details. These amounts of released copper (plus released zinc in the case of Cu5Zn5Al1Sn_ASW_) are in all cases sufficient for the complete killing of the bacteria, according to the kinetic curve for bacteria viability presented in Figure S6. Hence, adherent bacteria (*B. subtilis*) present on all investigated surfaces and interfaces described in Section 3.3 have prior to analysis undergone complete killing. In what follows, the underlying mechanisms of contact killing induced by Cu will be discussed in view of some key observations made including cell membrane damage, the formation of nanosized copper-containing particles, and intracellular copper redox reactions.

#### 3.4.1 Cell Membrane Damage

One of the most obvious observations of this study is the impairment of the cell membrane, as shown by TEM (Figures 4–6, S2, and S3). Cell membrane damage has been one of the most well-recognized mechanisms of contact killing, primarily induced by an extensive release of copper ions from the matrix.^17,45^ Yet, this is not the only explanation according to the literature.^20^ In addition to cell membrane damage, other copper-induced effects include peroxidation of membrane phospholipids caused by Cu surface contact or immersion in a Cu(II)-containing fluid. This has been pointed out to be responsible for copper alloy-mediated surface killing, ultimately leading to the loss of cell membrane integrity, DNA degradation, and cell death.^12,46^ In addition to the cell membrane ruptures caused by Cu surfaces and copper ions, other stress phenomena such as reactive oxygen species (ROS) may also result in loss of membrane integrity and in cytoplasmic content, thereby contributing to contact killing.^7,45^ Although the exact underlying biochemistry still remains unknown, it is clear that one of the major causes of Cu-induced contact killing is the damage of cell membranes, as clearly evidenced herein.

#### 3.4.2 Formation of Intracellular Nanosized Copper-Containing Particles

Another major observation is the formation of copper-rich nanosized particles in the cells and in the intact part of the cell membrane, as shown in Figures 4 and 5. To our knowledge, the direct observation of bacterial-induced intracellular formation of copper-rich nanoparticles has not been observed before. However, extracellular biogenic production of nanoparticles by, e.g., fungi, plant extracts, and bacteria, is frequently reported and often regarded as a more ecofriendly alternative to physicochemical production routes.^47^ Bacterial-induced synthesis of copper nanoparticles^48^ and copper oxide nanoparticles^49^ has also been reported, although not as often as of other metals such as gold and silver.^50,51^ A detailed mechanistic study of bacterial-induced gold (Au) nanoparticle production^49^ illustrated that the precursor for nanoparticle fabrication is Au(III) ions forming complexes with the bacterial cell wall constituents. This is followed by reduction of the metal ion by, e.g., proteins or enzymes on the cell wall, and transportation into the cytoplasm to form gold nanoparticles. Depending on the metal and the bacterium, a large variety of biomolecules in the bacteria have been reported as responsible for nanoparticle production, which can occur both intracellularly or extracellularly. However, nanoparticles may not only be formed in contact with constituents of the cell compartment but also as a result of interactions with the EPS.^52^

In accordance with our observations of copper nanoparticles within the bacteria, Santo et al. claimed that the level of copper ions remained high throughout the contact killing process, whereby intracellular copper leads to membrane and cell envelope damage.^53^ The exact mechanism for bacterial-induced copper nanoparticle formation cannot be deduced from this study, but two different reaction routes may be possible. One route is a consequence of the observed cell membrane damage, which may be responsible for the presence of larger particles observed next to the Cu substrate, see the TKD map in Figure 8. The images in Figure 8 suggest that the initial oxide on the Cu surface, predominantly Cu_2_O, is fragmentized upon contact with the bacteria and diffuses through the damaged cell membrane into the interior where it forms nanoparticles. Such a process has in the literature been regarded as a bacterial defense to withstand toxic effects of copper ions.^46^ The other reaction route is based on dissolved copper ions, which enter through the ruptured cell membrane into the interior of the bacterium. Similar to the first reaction route, the formation of copper-containing nanoparticles upon interactions between the bacteria and copper ions may be regarded as a detoxification mechanism^50^ in which more soluble cuprous ions are transformed within the bacteria into less soluble or even insoluble nanosized cupric features.

The production of copper-containing nanoparticles could be one example of how bacteria develop specific homeostasis systems that can act as defense systems to maintain cellular metabolism at different ambient copper concentrations.^54−56^ Other examples are cytoplasmic proteins, which can bind, sequester, or store metals,^57,58^ also bacterial-induced transformation of metals to metal oxides, metal sulfides, metal–protein aggregates, or elemental metal crystals, which form particulates that are closely associated with the cytoplasmic membrane.^59^ Hence, the crystalline copper oxide nanoparticles found within the cells by HRTEM (Figures 7 and S4) and TKD (Figure 8) may be regarded as a way for the bacteria to enhance their resistance against copper. This is a topic that has barely been studied so far with respect to contact killing,^7^ and awaits further exploration.

#### 3.4.3 Intracellular Copper Redox Reactions

Under atmospheric corrosion of simulated high-touch conditions, the formed corrosion products are predominately composed of Cu_2_O and to a minor extent of CuCl/Cu_2_(OH)Cl upon exposure with predeposited ASW.^20,23^ Thus, the dissolution of copper ions from the surfaces and their entrance via the damaged membrane into the bacteria would primarily be as cuprous ions, Cu(I), and/or fragmentized Cu_2_O species, which possess more effective antimicrobial properties than cupric ions, Cu(II).^60^ However, most of nanosized particles characterized by HRTEM within the cells were identified as CuO rather than as a Cu(I)-compound (Figures 7 and S4), implying the occurrence of redox reactions within the cells. Species involved in intracellular redox reactions may either be produced by the bacteria (endogenous) or, such as in this case, originate from the surrounding environment (exogenous). Examples of reactive species involved in such redox reactions are hydrogen peroxide (H_2_O_2_), the hydroxyl radical (OH^•^), and the superoxide anion (O_2_^–^).^61^ It is well known that the redox reaction between cuprous Cu(I) and cupric Cu(II) ions can lead to the generation of reactive hydroxyl radicals (OH^•^) in a Fenton-type reaction (see below) that is detrimental to cellular molecules, such as DNA, proteins, and lipids.^7,45,62^ Yet this reaction may not be the primary toxic mechanism, although often claimed to be, because of the ability of the cells to retain hydrogen peroxide (H_2_O_2_) at low levels at the expense of hydroxyl radicals.^7^
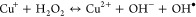
1The oxidation of Cu(I) to Cu(II), on the other hand, could be one of the means of the bacteria to better resist copper toxicity by converting Cu(I) to less toxic Cu(II).^58^ This chemical modification process can create metal crystal precipitates or generate organometallic small-molecule colloids/complexes,^46^ which may act as precursors to the formation of intracellular biosynthesis of copper-containing nanoparticles, as discussed in Section 3.4.2.

**Figure 9:**
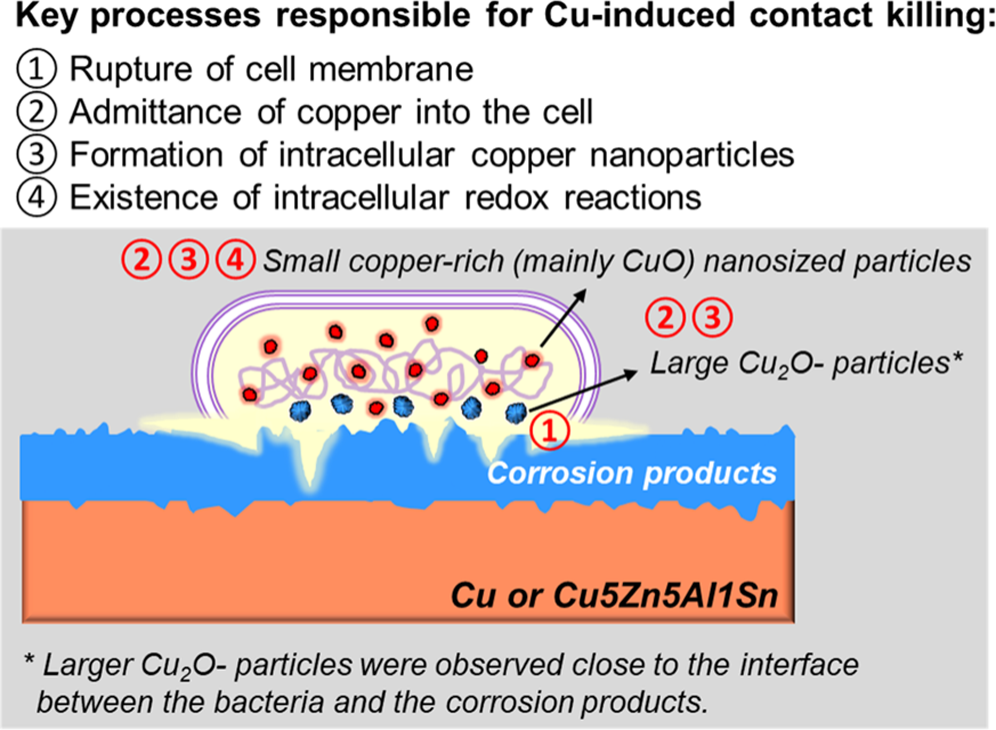
Schematic illustration of the findings at the bioinorganic interface between a bacterium and the differently corroded surfaces of Cu, Cu_ASW,_ and Cu5Zn5Al1Sn_ASW_ to mimic contact killing by copper.

In all, the experimental findings and conclusions deduced from analysis of the bioinorganic interfaces of Cu/Cu5Zn5Al1Sn with different extents of corrosion in contact with *B. subtilis* are schematically illustrated in Figure 9. Cell membrane rupture leads to the ingress of copper ions or fragmentized Cu_2_O particles into the cell once in contact with the Cu and Cu5Zn5Al1Sn surface, resulting in the formation of copper-rich nanosized particles within the bacteria. The crystalline structure and chemical speciation of these particles suggest intracellular redox reactions between cuprous and cupric ions and the formation of organometallic species/complexes. This may occur either inside or outside the cell.

## 4 Conclusions

The mechanism of bacterial contact killing induced by copper was explored through high-resolution studies by means of FIB-SEM, TEM, STEM, and nano-FTIR microscopy of individual bacteria of *B. subtilis* in direct contact with Cu metal and Cu5Zn5Al1Sn surfaces. This approach permitted subcellular information to be extracted from the bioinorganic interface between a single bacterium and the Cu/Cu5Zn5Al1Sn surfaces. The following conclusions could be drawn of relevance for contact killing:

(1) Early stages of interaction between individual bacteria and the metal/alloy substrate include cell leakage of extracellular polymeric substances (EPS) from the bacterium and changes in matrix surface composition upon adherence of bacteria.
(2) Direct evidence of cell membrane damage was observed upon contact between the bacteria and the Cu or Cu5Zn5Al1Sn surfaces.
(3) Cell membrane damage allowed copper to enter into the bacterial cells either as small fragmentized Cu_2_O particles or as copper ions dissolved from the corrosion products.
(4) Intracellular formation of copper-containing nanoparticles was higher in the bacterium in contact with Cu metal than with Cu5Zn5Al1Sn, related to more corroded surfaces and a higher copper release rate of Cu metal than of Cu5Zn5Al1Sn.
(5) The nanosized particles consist primarily of CuO with smaller amounts of Cu_2_O, suggesting that intracellular redox reactions between cuprous and cupric ions are important.
(6) The formation of nanoparticles containing primarily CuO may be regarded as a possible detoxification mechanism against copper-induced contact killing.

## Supporting Information Available

Corrosion product layer presented by the SEM image of the cross-sectional morphology of the Cu surface with predeposited ASW (Figure S1), morphology and elemental distribution of the interfaces between the bacterium and the Cu surface without predeposited ASW (Figure S2) as well as the bacterium and the Cu5Zn5Al1Sn surface with predeposited ASW (Figure S3) presented by STEM, crystalline nanosized copper-enriched particles within the bacterium in contact with Cu and Cu5Zn5Al1Sn with predeposited ASW demonstrated by HRTEM (Figure S4), morphological and microstructural information of the interface between the bacterium and the Cu5Zn5Al1Sn surface with predeposited ASW showed by SEM and TKD (Figure S5), and rapid killing rate of Cu and Cu5Zn5Al1Sn with and without predeposited ASW in contact with bacteria (Figure S6) (PDF)

## References

[ref1] Şen Karaman D.; Ercan U. K.; Bakay E.; Topaloğlu N.; Rosenholm J. M. Evolving Technologies and Strategies for Combating Antibacterial Resistance in the Advent of the Postantibiotic Era. Adv. Funct. Mater. 2020, 30, 1908783 10.1002/adfm.201908783.

[ref2] O’Neill J. Review on Antimicrobial Resistance: Tackling Drug-Resistant Infections Globally: Final Report and Recommendations: The Review on Microbial Resistance; Wellcome Trust, 2016; p 80.

[ref3] Singh A.; Gautam P. K.; Verma A.; Singh V.; Shivapriya P. M.; Shivalkar S.; Sahoo A. K.; Samanta S. K. Green Synthesis of Metallic Nanoparticles as Effective Alternatives to Treat Antibiotics Resistant Bacterial Infections: A Review. Biotechnol. Rep. 2020, 25, e00427 10.1016/j.btre.2020.e00427. PMC7005563 32055457

[ref4] van Doremalen N.; Bushmaker T.; Morris D. H.; Holbrook M. G.; Gamble A.; Williamson B. N.; Tamin A.; Harcourt J. L.; Thornburg N. J.; Gerber S. I. Aerosol and Surface Stability of Sars-Cov-2 as Compared with Sars-Cov-1. N. Engl. J. Med. 2020, 382, 1564–1567. 10.1056/NEJMc2004973. 32182409 PMC7121658

[ref5] Hutasoit N.; Kennedy B.; Hamilton S.; Luttick A.; Rashid R. A. R.; Palanisamy S. Sars-Cov-2 (Covid-19) Inactivation Capability of Copper-Coated Touch Surface Fabricated by Cold-Spray Technology. Manuf. Lett. 2020, 25, 93–97. 10.1016/j.mfglet.2020.08.007. 32904558 PMC7455544

[ref6] Behzadinasab S.; Chin A.; Hosseini M.; Poon L.; Ducker W. A. A Surface Coating That Rapidly Inactivates Sars-Cov-2. ACS Appl. Mater. Interfaces 2020, 12, 34723–34727. 10.1021/acsami.0c11425. 32657566 PMC7385996

[ref7] Grass G.; Rensing C.; Solioz M. Metallic Copper as an Antimicrobial Surface. Appl. Environ. Microbiol. 2011, 77, 1541–1547. 10.1128/AEM.02766-10. 21193661 PMC3067274

[ref8] Michels H. T.; Michels C. A. Copper Alloys-the New ‘Old’weapon in the Fight against Infectious Disease. Microbiology 2016, 10, 23–45.

[ref9] Muller M. P.; MacDougall C.; Lim M.; Armstrong I.; Bialachowski A.; Callery S.; Ciccotelli W.; Cividino M.; Dennis J.; Hota S. Antimicrobial Surfaces to Prevent Healthcare-Associated Infections: A Systematic Review. J. Hosp. Infect. 2016, 92, 7–13. 10.1016/j.jhin.2015.09.008. 26601608

[ref10] Schmidt M. G.; Tuuri R. E.; Dharsee A.; Attaway H. H.; Fairey S. E.; Borg K. T.; Salgado C. D.; Hirsch B. E. Antimicrobial Copper Alloys Decreased Bacteria on Stethoscope Surfaces. Am. J. Infect. Control 2017, 45, 642–647. 10.1016/j.ajic.2017.01.030. 28302430

[ref11] U.S. Environmental Protection Agency (EPA). EPA Registers Copper-Containing Alloy Products. 2008, http://www.epa.gov/pesticides/factsheets/copper-alloy-products.htm.

[ref12] Hong R.; Kang T. Y.; Michels C. A.; Gadura N. Membrane Lipid Peroxidation in Copper Alloy-Mediated Contact Killing of *Escherichia coli*. Appl. Environ. Microbiol. 2012, 78, 1776–1784. 10.1128/AEM.07068-11. 22247141 PMC3298164

[ref13] Vincent M.; Duval R. E.; Hartemann P.; Engels-Deutsch M. Contact Killing and Antimicrobial Properties of Copper. J. Appl. Microbiol. 2018, 124, 1032–1046. 10.1111/jam.13681. 29280540

[ref14] Rosenberg M.; Vija H.; Kahru A.; Keevil C. W.; Ivask A. Rapid in Situ Assessment of Cu-Ion Mediated Effects and Antibacterial Efficacy of Copper Surfaces. Sci. Rep. 2018, 8, 8172 10.1038/s41598-018-26391-8. 29802355 PMC5970231

[ref15] Gross T. M.; Lahiri J.; Golas A.; Luo J.; Verrier F.; Kurzejewski J. L.; Baker D. E.; Wang J.; Novak P. F.; Snyder M. J. Copper-Containing Glass Ceramic with High Antimicrobial Efficacy. Nat. Commun. 2019, 10, 1979 10.1038/s41467-019-09946-9. 31040286 PMC6491652

[ref16] Zeiger M.; Solioz M.; Edongué H.; Arzt E.; Schneider A. S. Surface Structure Influences Contact Killing of Bacteria by Copper. MicrobiologyOpen 2014, 3, 327–332. 10.1002/mbo3.170. 24740976 PMC4082706

[ref17] Li M.; Ma Z.; Zhu Y.; Xia H.; Yao M.; Chu X.; Wang X.; Yang K.; Yang M.; Zhang Y.; Mao C. Toward a Molecular Understanding of the Antibacterial Mechanism of Copper-Bearing Titanium Alloys against Staphylococcus Aureus. Adv. Healthcare Mater. 2016, 5, 557–566. 10.1002/adhm.201500712. PMC4785048 26692564

[ref18] Warnes S. L.; Keevil C. W. Mechanism of Copper Surface Toxicity in Vancomycin-Resistant Enterococci Following Wet or Dry Surface Contact. Appl. Environ. Microbiol. 2011, 77, 6049–6059. 10.1128/AEM.00597-11. 21742916 PMC3165410

[ref19] Leygraf C.; Odnevall Wallinder I.; Tidblad J.; Graedel T. Atmospheric Corrosion, 2nd ed.; John Wiley & Sons, 2016.

[ref20] Chang T.; Sepati M.; Herting G.; Leygraf C.; Rajarao G. K.; Butina K.; Richter-Dahlfors A.; Blomberg E.; Odnevall Wallinder I. A Novel Methodology to Study Antimicrobial Properties of High-Touch Surfaces Used for Indoor Hygiene Applications—a Study on Cu Metal. PLoS One 2021, 16, e0247081 10.1371/journal.pone.0247081. 33630868 PMC7906481

[ref21] Mikolay A.; Huggett S.; Tikana L.; Grass G.; Braun J.; Nies D. H. Survival of Bacteria on Metallic Copper Surfaces in a Hospital Trial. Appl. Microbiol. Biotechnol. 2010, 87, 1875–1879. 10.1007/s00253-010-2640-1. 20449737

[ref22] Walkowicz M.; Osuch P.; Smyrak B.; Knych T.; Rudnik E.; Cieniek Ł.; Różańska A.; Chmielarczyk A.; Romaniszyn D.; Bulanda M. Impact of Oxidation of Copper and Its Alloys in Laboratory-Simulated Conditions on Their Antimicrobial Efficiency. Corros. Sci. 2018, 140, 321–332. 10.1016/j.corsci.2018.05.033.

[ref23] Chang T.; Butina K.; Herting G.; Rajarao G. K.; Richter-Dahlfors A.; Blomberg E.; Odnevall Wallinder I.; Leygraf C. The Interplay between Atmospheric Corrosion and Antimicrobial Efficiency of Cu and Cu5zn5al1sn During Simulated High-Touch Conditions. Corros. Sci. 2021, 185, 109433 10.1016/j.corsci.2021.109433.

[ref24] Chang T.; Odnevall Wallinder I.; Jin Y.; Leygraf C. The Golden Alloy Cu-5zn-5al-1sn: A Multi-Analytical Surface Characterization. Corros. Sci. 2018, 131, 94–103. 10.1016/j.corsci.2017.11.014.

[ref25] Chang T.; Herting G.; Jin Y.; Leygraf C.; Odnevall Wallinder I. The Golden Alloy Cu-5zn-5al-1sn: Patina Evolution in Chloride-Containing Atmospheres. Corros. Sci. 2018, 133, 190–203. 10.1016/j.corsci.2018.01.027.

[ref26] Kearns D. B.; Chu F.; Branda S. S.; Kolter R.; Losick R. A Master Regulator for Biofilm Formation by *Bacillus subtilis*. Mol. Microbiol. 2005, 55, 739–749. 10.1111/j.1365-2958.2004.04440.x. 15661000

[ref27] Hall-Stoodley L.; Costerton J. W.; Stoodley P. Bacterial Biofilms: From the Natural Environment to Infectious Diseases. Nat. Rev. Microbiol. 2004, 2, 95–108. 10.1038/nrmicro821. 15040259

[ref28] Aurubis Stolberg, 2021. https://www.aurubis-stolberg.com/wdb/band/eng/Copper/Cu-DHP-PNA%20219_EN.

[ref29] European Committee for Standardisation (CEN). Reference Test Method for Release of Nickel from All Post Assemblies Which Are Inserted into Pierced Parts of the Human Body and Articles Intended to Come into Direct and Prolonged Contact with the Skin; EN 1811:2011; European Committee for Standardisation (CEN), 2011.

[ref30] Zhang X.; Leygraf C.; Odnevall Wallinder I. Atmospheric Corrosion of Galfan Coatings on Steel in Chloride-Rich Environments. Corros. Sci. 2013, 73, 62–71. 10.1016/j.corsci.2013.03.025.

[ref31] Zhao W.; Johnson C. M. Perspective—Nano Infrared Microscopy: Obtaining Chemical Information on the Nanoscale in Corrosion Studies. J. Electrochem. Soc. 2019, 166, C3456 10.1149/2.0531911jes.

[ref32] Nečas D.; Klapetek P. Gwyddion: An Open-Source Software for Spm Data Analysis. Open Phys. 2012, 10, 181–188. 10.2478/s11534-011-0096-2.

[ref33] Amenabar I.; Poly S.; Nuansing W.; Hubrich E. H.; Govyadinov A. A.; Huth F.; Krutokhvostov R.; Zhang L.; Knez M.; Heberle J. Structural Analysis and Mapping of Individual Protein Complexes by Infrared Nanospectroscopy. Nat. Commun. 2013, 4, 2890 10.1038/ncomms3890. 24301518 PMC3863900

[ref34] Garip S.; Gozen A. C.; Severcan F. Use of Fourier Transform Infrared Spectroscopy for Rapid Comparative Analysis of Bacillus and Micrococcus Isolates. Food Chem. 2009, 113, 1301–1307. 10.1016/j.foodchem.2008.08.063.

[ref35] Giotta L.; Mastrogiacomo D.; Italiano F.; Milano F.; Agostiano A.; Nagy K.; Valli L.; Trotta M. Reversible Binding of Metal Ions onto Bacterial Layers Revealed by Protonation-Induced ATR-FTIR Difference Spectroscopy. Langmuir 2011, 27, 3762–3773. 10.1021/la104868m. 21395289

[ref36] Omoike A.; Chorover J. Spectroscopic Study of Extracellular Polymeric Substances from Bacillus S Ubtilis: Aqueous Chemistry and Adsorption Effects. Biomacromolecules 2004, 5, 1219–1230. 10.1021/bm034461z. 15244434

[ref37] Desvaux M.; Dumas E.; Chafsey I.; Hebraud M. Protein Cell Surface Display in Gram-Positive Bacteria: From Single Protein to Macromolecular Protein Structure. FEMS Microbiol. Lett. 2006, 256, 1–15. 10.1111/j.1574-6968.2006.00122.x. 16487313

[ref38] Badireddy A. R.; Korpol B. R.; Chellam S.; Gassman P. L.; Engelhard M. H.; Lea A. S.; Rosso K. M. Spectroscopic Characterization of Extracellular Polymeric Substances from *Escherichia coli* and *Serratia marcescens*: Suppression Using Sub-Inhibitory Concentrations of Bismuth Thiols. Biomacromolecules 2008, 9, 3079–3089. 10.1021/bm800600p. 18937399

[ref39] Zhang P.; Chen Y.-P.; Peng M.-W.; Guo J.-S.; Shen Y.; Yan P.; Zhou Q.-H.; Jiang J.; Fang F. Extracellular Polymeric Substances Dependence of Surface Interactions of *Bacillus subtilis* with Cd^2+^ and Pb^2+^: An Investigation Combined with Surface Plasmon Resonance and Infrared Spectra. Colloids Surf., B 2017, 154, 357–364. 10.1016/j.colsurfb.2017.03.046. 28365425

[ref40] Chug R.; Gour V. S.; Mathur S.; Kothari S. Optimization of Extracellular Polymeric Substances Production Using Azotobacter Beijreinckii and *Bacillus subtilis* and Its Application in Chromium (Vi) Removal. Bioresour. Technol. 2016, 214, 604–608. 10.1016/j.biortech.2016.05.010. 27183236

[ref41] Madigan M. T.; Martinko J. M.; Dunlap P. V.; Clark D. P. Brock Biology of Microorganisms 12th Edn. Int. Microbiol. 2008, 11, 65–73.

[ref42] Allen C.; Loo J. F.; Yu S.; Kong S.; Chan T.-F. Monitoring Bacterial Growth Using Tunable Resistive Pulse Sensing with a Pore-Based Technique. Appl. Microbiol. Biotechnol. 2014, 98, 855–862. 10.1007/s00253-013-5377-9. 24287933

[ref43] Hogan C. M. Bacteria. In Encyclopedia of Earth; National Council for Science and the Environment: Washington, DC, 2010.

[ref44] Macomber L.; Imlay J. A. The Iron-Sulfur Clusters of Dehydratases Are Primary Intracellular Targets of Copper Toxicity. Proc. Natl. Acad. Sci. U.S.A. 2009, 106, 8344–8349. 10.1073/pnas.0812808106. 19416816 PMC2688863

[ref45] Santo C. E.; Taudte N.; Nies D. H.; Grass G. Contribution of Copper Ion Resistance to Survival of *Escherichia coli* on Metallic Copper Surfaces. Appl. Environ. Microbiol. 2008, 74, 977–986. 10.1128/AEM.01938-07. 18156321 PMC2258564

[ref46] Lemire J. A.; Harrison J. J.; Turner R. J. Antimicrobial Activity of Metals: Mechanisms, Molecular Targets and Applications. Nat. Rev. Microbiol. 2013, 11, 371–384. 10.1038/nrmicro3028. 23669886

[ref47] Popescu M.; Velea A.; Lőrinczi A. Biogenic Production of Nanoparticles. Dig. J. Nanomater. Biostruct. 2010, 5, 1035–1040.

[ref48] de Andrade C. J.; De Andrade L. M.; Mendes M. A.; Do C. A. O. An Overview on the Production of Microbial Copper Nanoparticles by Bacteria, Fungi and Algae. Global J. Res. Eng. 2017, 17, 1568.

[ref49] Waris A.; Din M.; Ali A.; Ali M.; Afridi S.; Baset A.; Khan A. U. A Comprehensive Review of Green Synthesis of Copper Oxide Nanoparticles and Their Diverse Biomedical Applications. Inorg. Chem. Commun. 2020, 123, 108369 10.1016/j.inoche.2020.108369.

[ref50] Das S. K.; Liang J.; Schmidt M.; Laffir F.; Marsili E. Biomineralization Mechanism of Gold by Zygomycete Fungi Rhizopous Oryzae. ACS Nano 2012, 6, 6165–6173. 10.1021/nn301502s. 22708541

[ref51] Klaus T.; Joerger R.; Olsson E.; Granqvist C.-G. Silver-Based Crystalline Nanoparticles, Microbially Fabricated. Proc. Natl. Acad. Sci. U.S.A. 1999, 96, 13611–13614. 10.1073/pnas.96.24.13611. 10570120 PMC24112

[ref52] Kang F.; Qu X.; Alvarez P. J.; Zhu D. Extracellular Saccharide-Mediated Reduction of Au3+ to Gold Nanoparticles: New Insights for Heavy Metals Biomineralization on Microbial Surfaces. Environ. Sci. Technol. 2017, 51, 2776–2785. 10.1021/acs.est.6b05930. 28151654

[ref53] Santo C. E.; Lam E. W.; Elowsky C. G.; Quaranta D.; Domaille D. W.; Chang C. J.; Grass G. Bacterial Killing by Dry Metallic Copper Surfaces. Appl. Environ. Microbiol. 2011, 77, 794–802. 10.1128/AEM.01599-10. 21148701 PMC3028699

[ref54] Rademacher C.; Masepohl B. Copper-Responsive Gene Regulation in Bacteria. Microbiology 2012, 158, 2451–2464. 10.1099/mic.0.058487-0. 22918892

[ref55] Dupont C. L.; Grass G.; Rensing C. Copper Toxicity and the Origin of Bacterial Resistance—New Insights and Applications. Metallomics 2011, 3, 1109–1118. 10.1039/c1mt00107h. 21984219

[ref56] Solioz M. Copper and Bacteria: Evolution, Homeostasis and Toxicity; Springer, 2018.

[ref57] Carrondo M. A. Ferritins, Iron Uptake and Storage from the Bacterioferritin Viewpoint. EMBO J. 2003, 22, 1959–1968. 10.1093/emboj/cdg215. 12727864 PMC156087

[ref58] Silver S.; Phung L. T. Bacterial Heavy Metal Resistance: New Surprises. Annu. Rev. Microbiol. 1996, 50, 753–789. 10.1146/annurev.micro.50.1.753. 8905098

[ref59] Zannoni D.; Borsetti F.; Harrison J. J.; Turner R. J. The Bacterial Response to the Chalcogen Metalloids Se and Te. Adv. Microb. Physiol. 2007, 53, 1–312. 10.1016/S0065-2911(07)53001-8. 17707143

[ref60] Hans M.; Erbe A.; Mathews S.; Chen Y.; Solioz M.; Mucklich F. Role of Copper Oxides in Contact Killing of Bacteria. Langmuir 2013, 29, 16160–16166. 10.1021/la404091z. 24344971

[ref61] Reniere M. L. Reduce, Induce, Thrive: Bacterial Redox Sensing During Pathogenesis. J. Bacteriol. 2018, 200, e00128-18 10.1128/JB.00128-18. 29891640 PMC6088161

[ref62] Yoshida Y.; Furuta S.; Niki E. Effects of Metal Chelating Agents on the Oxidation of Lipids Induced by Copper and Iron. Biochim. Biophys. Acta, Lipids Lipid Metab. 1993, 1210, 81–88. 10.1016/0005-2760(93)90052-B. 8257723

