# Supplementary material for: High-Resolution Microscopical Studies of Contact Killing Mechanisms on Copper-Based Surfaces
Source: ACS Appl Mater Interfaces. 2021 Oct 7;13(41):49402–13. doi: 10.1021/acsami.1c11236 (PMC8532116; doi:10.1021/acsami.1c11236)
Supplement: Supplementary file 1 — am1c11236_si_001.pdf [file am1c11236_si_001.pdf]

Supplementary information

High resolution microscopical studies of contact killing mechanisms on copper-based surfaces

Tingru Chang[a,b,c*], R. Prasath Babu[d], Weijie Zhao[a], C. Magnus Johnson[a], Peter Hedström[d], Inger Odnevall[a,b,c], Christofer Leygraf[a]

[a] KTH Royal Institute of Technology, Department of Chemistry, Div. Surface and Corrosion Science, Drottning Kristinas väg 51, SE-100 44 Stockholm, Sweden

[b] AIMES - Center for the Advancement of Integrated Medical and Engineering Sciences at Karolinska Institutet and KTH Royal Institute of Technology, SE-171 77 Stockholm, Sweden

[c] Department of Neuroscience, Karolinska Institutet, SE-171 77 Stockholm, Sweden

[d] KTH Royal Institute of Technology, Department of Materials Science and Engineering, SE-100 44 Stockholm, Sweden

***Corresponding author, e-mail: tingru@kth.se**

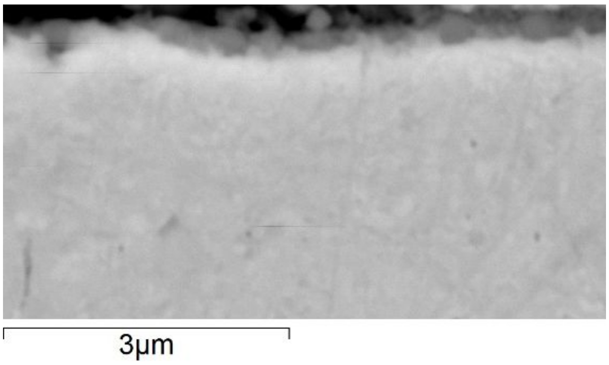

Figure S1. SEM image of the cross-section morphology of Cu_{ASW} (Cu with pre-deposited ASW).

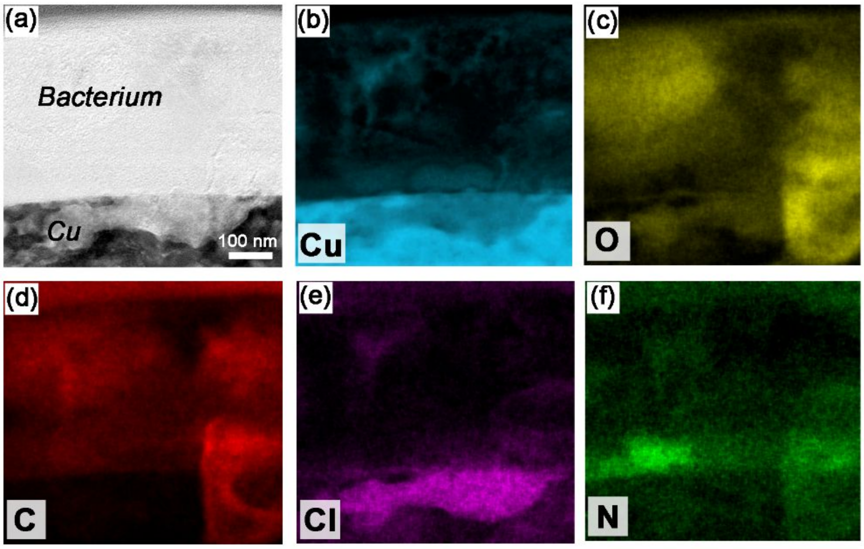

Figure S2. STEM image (a) of morphology at the interfaces of Pt protection coating, bacterium, and Cu metal surface (up to down) and the corresponding STEM-EDS mapping of Cu (b), O (c), C (d), Cl (e), and N (f).

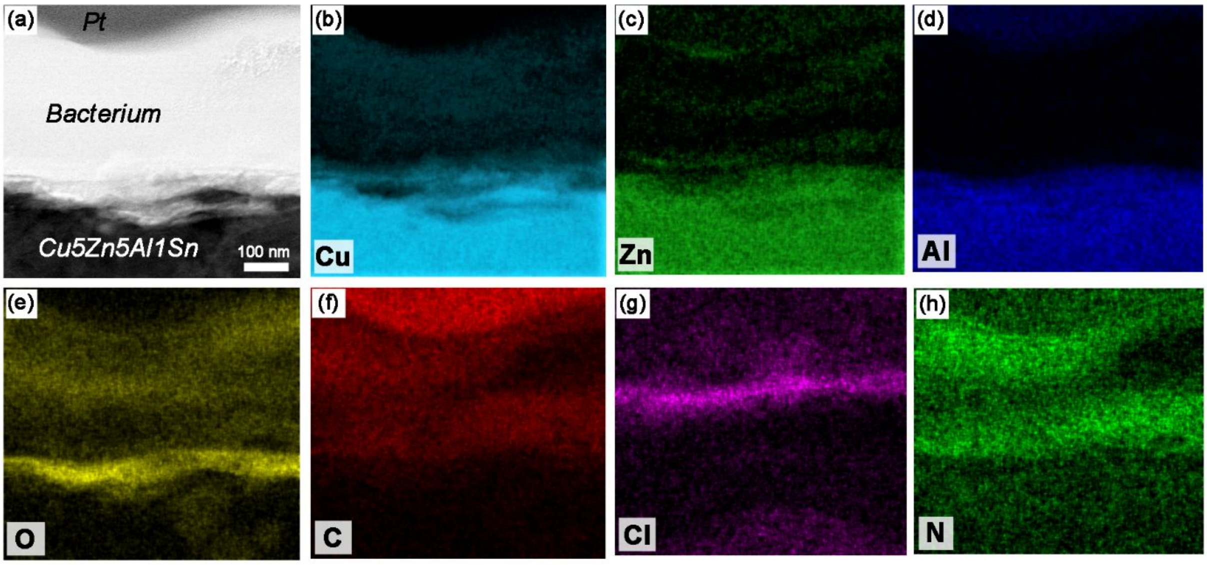

Figure S3. STEM image (a) of morphology at the interfaces between bacterium and the $Cu5Zn5Al1Sn_{ASW}$ surface (left on the top) and the corresponding STEM-EDS mapping of Cu (b), Zn (c), Al (d), O (e), C (f), Cl (g), and N (h).

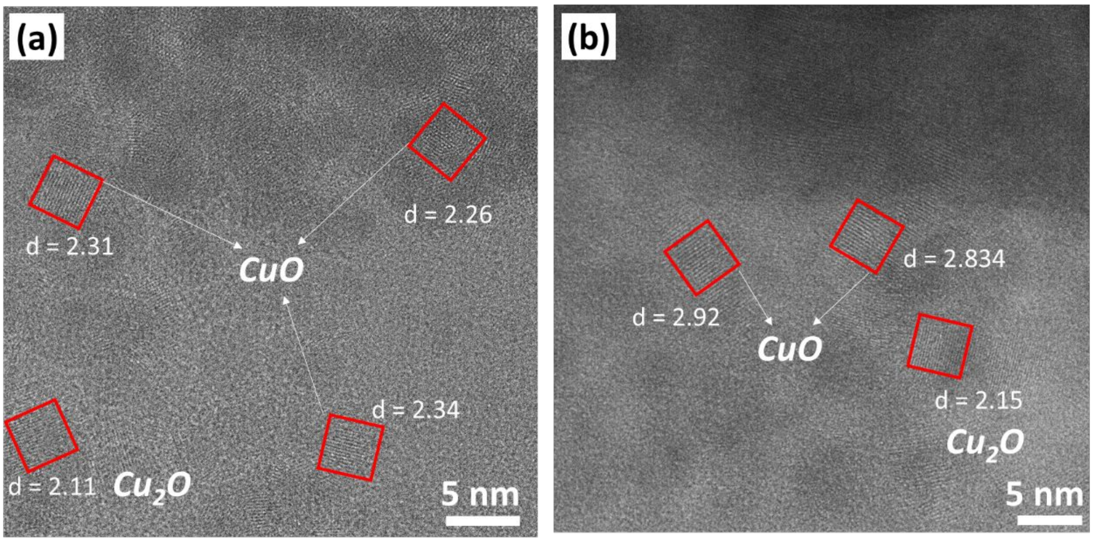

Figure S4. HRTEM image of the copper-enriched particles observed within the bacterium in contact with surfaces of Cu_{ASW} (a) and $Cu5Zn5Al1Sn_{ASW}$ (b).

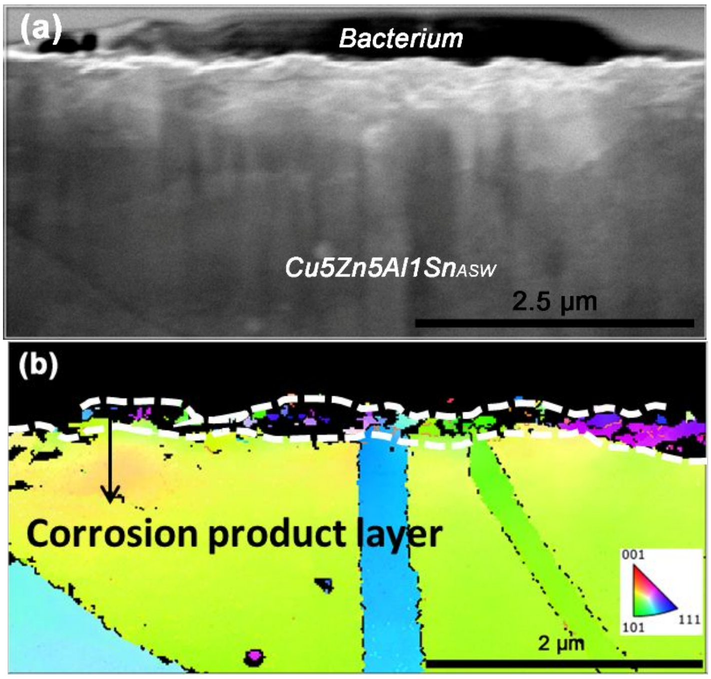

Figure S5. SEM image of the interfaces between the Pt protection coating, and bacterium, and Cu5Zn5Al1SnASW (up to down) obtained by FIB (a) and the corresponding inverse pole figure (IPF) map (b). The white dashed lines indicate the boundaries of the corrosion product layer in between the bacterium and the substrate.

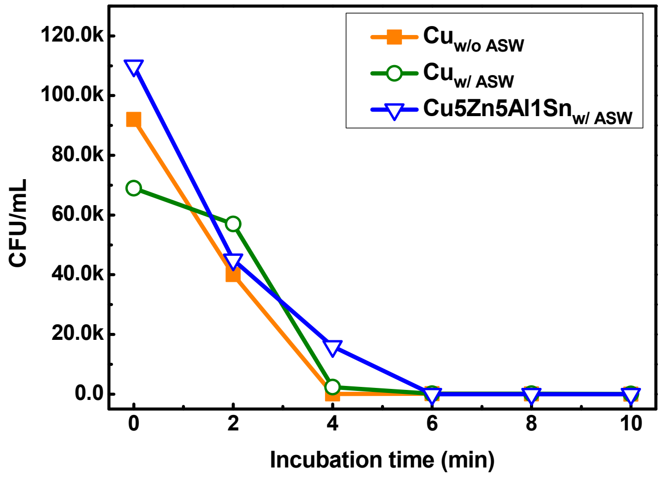

Figure S6. Kinetics of bacterial viability with an average standard deviation of 30% (not indicated in the graph) on the 1 day exposed surfaces of Cu and Cu5Zn5Al1Sn with and without pre-deposited ASW. Reproduced with permission from reference 23 in-text references. Copyright © 1969, Elsevier.

Reference

(23) Chang, T.; Butina, K.; Herting, G.; Rajarao, G. K.; Richter-Dahlfors, A.; Blomberg, E.; Odnevall Wallinder, I.; Leygraf, C. The Interplay between Atmospheric Corrosion and Antimicrobial Efficiency of Cu and Cu5zn5al1sn During Simulated High-Touch Conditions. *Corros. Sci.* **2021,** *185*, 109433.
